# Biomarker repurposing: Therapeutic drug monitoring of serum theophylline offers a potential diagnostic biomarker of Parkinson’s disease

**DOI:** 10.1371/journal.pone.0201260

**Published:** 2018-07-25

**Authors:** Takuma Ohmichi, Takashi Kasai, Tadashi Kosaka, Keisuke Shikata, Harutsugu Tatebe, Ryotaro Ishii, Makiko Shinomoto, Toshiki Mizuno, Takahiko Tokuda

**Affiliations:** 1 Department of Neurology, Kyoto Prefectural University of Medicine, Kyoto, Japan; 2 Department of Pharmacy, University Hospital, Kyoto Prefectural University of Medicine, Kyoto, Japan; 3 Department of Zaitaku (Homecare) Medicine, Kyoto Prefectural University of Medicine, Kyoto, Japan; 4 North Medical Center, Kyoto Prefectural University of Medicine, Kyoto, Japan; 5 Department of Molecular Pathobiology of Brain Diseases, Kyoto Prefectural University of Medicine, Kyoto, Japan; 6 AMED-CREST, Japan Agency for Medical Research and Development, Tokyo, Japan; Osaka University Graduate School of Medicine, JAPAN

## Abstract

Caffeine has been considered a neuroprotective agent against Parkinson’s disease (PD). Recent metabolomic analysis showed that levels of caffeine and its metabolites were decreased in sera from patients with PD compared with those from healthy controls. We focused on theophylline, which is one of the primary caffeine metabolites, as a candidate biomarker of PD because: (1) its serum level can be measured in hospital laboratories by standardized immunoassay kits for therapeutic drug monitoring and (2) because it is less markedly affected by caffeine intake. This was a pilot study to measure the levels of theophylline in sera of 31 patients with PD and 33 age-matched disease controls using an immunoassay kit. We confirmed the previous finding of significantly lower levels of serum theophylline in the PD group compared with control group (PD: 0.07±0.09 μg/mL, control: 0.18±0.24 μg/mL, p<0.05). Using such an approach of applying known medical biomarkers for neurodegenerative diseases may allow us to skip the process from the discovery phase to clinical application, and subsequently shorten the period of time necessary for biomarker development.

## Introduction

Parkinson’s disease (PD) is the second most common neurodegenerative disorder after Alzheimer’s disease (AD). There is no drug or treatment strategy proven to be neuroprotective or disease-modifying for PD patients. To achieve efficient disease-modification in the early phase of the disease, biomarkers that aid with the presymptomatic diagnosis of PD are critical. Nuclear radiological biomarkers with isotopes including ^123^I-Ioflupane have been widely applied for the diagnosis of clinical PD, which clinically manifests as motor symptoms due to the loss of dopaminergic neurons. Furthermore, these biomarkers are expected to undergo future development for clinical application to individuals at risk of PD, who are regarded as having prodromal PD, presenting solely with early signs or non-motor symptoms of the disease [1, 2]. On the other hand, some chemicals in cerebrospinal fluid (CSF) may also have diagnostic value for the detection of neurodegenerative process. For example, several case-control studies generated consistent findings whereby abnormally elevated oligomeric α-synuclein in CSF can be observed in patients with clinical PD [3, 4]. However, those approaches cannot be practically utilized due to being expensive, highly invasive, or labor-intensive procedures. In other words, there remains an urgent need to develop blood-based biomarkers that are more cost-efficient and less-invasive to screen for preclinical PD patients, who show neurodegeneration in the absence of any specific non-motor or motor symptoms of PD, in a general population [5].

Caffeine and its metabolites have been considered as candidate blood-based biomarkers of PD. One of the reasons is based on metabolomic analyses, revealing that serum levels of caffeine metabolites in patients with PD are significantly lower compared with those in controls and that their decreased plasma levels in patients with early PD are correlated with disease progression [6, 7]. Recently, such findings were validated in a cohort comprising a sufficient number of subjects from a single institute by the same group [8]. Considering the reports to date, multi-institutional validation studies should be planned as the next step to facilitate the clinical application of caffeine metabolites as diagnostic and/or progression biomarkers of PD [8]. However, the quantification method using mass-spectrometry in these studies might not be suitable for such multi-institutional validation or for general medical practice due to the cost of running the devices and difficulty of maintaining inter-assay and inter-laboratory variance low enough for precision performance.

In this study, we focused on the diagnostic value of serum theophylline for PD, one of the primary caffeine metabolites, for the following reasons. First, measurements of serum theophylline levels have been commonly employed for therapeutic drug monitoring in patients with bronchial asthma and have already been adequately standardized to manufacture commercially available immunoassay kits. Second, serum levels of theophylline are less affected by caffeine intake compared with other primary caffeine metabolites, such as paraxanthine and theobromine [9]. Taken together, serum levels of theophylline represent caffeine metabolism without being affected by food intake; therefore, it can potentially serve as a blood-based biomarker of PD with acceptable inter-assay and inter-laboratory reproducibility. Herein, we conducted this pilot study to examine serum levels of theophylline measured by one of the commercially available immunoassay kits. The aim of this study was to confirm the difference between patients with PD and controls observed in previous studies using mass-spectrometry.

## Materials and methods

### Study design, ethics statement, and subject recruitment

We recruited 31 patients with PD (PD group) and 33 age-matched controls (control group). All study subjects provided written informed consent before participation and the study protocols were approved by the Kyoto Prefectural University of Medicine Ethics Committee (reference numbers: RBMR-C-559 for the PD group and ERG-G12-3 for the control group). Informed consent was obtained from the subjects when possible, or from the nearest relative when not possible. The study procedures were designed and performed in accordance with the Declaration of Helsinki. Patients were eligible for inclusion if they had been diagnosed with PD based on the UK PD Society Brain Bank criteria [10]. Subjects were excluded if they had been administered theophylline or other xanthine derivatives. The age-matched control subjects comprised 33 disease controls, comprising those with cranial and peripheral neuropathy (n = 11), cervical spondylosis (n = 11), myopathy (n = 3), disuse syndrome (n = 2), benign positional vertigo (n = 1), idiopathic intracranial hypertension (n = 1), hyponatremia (n = 1), Asperger syndrome (n = 1), head drop syndrome (n = 1), and dysarthria (n = 1). Serum samples were obtained via venous puncture under resting conditions after three-hour fasting in the hospital. After collection, serum was separated by centrifugation for 15 min at 2,000 g and then stored at -80°C until analysis. The clinical data, including Hoehn & Yahr (H&Y) stages, Unified Parkinson’s Disease Rating Scale motor section (UPDRS-III) scores, Mini-Mental State Examination (MMSE) score, and heart/mediastinum (H/M) ratio in the early and delayed phases of myocardial imaging with ^123^l-metaiodobenzylguanidine (MIBG) were evaluated within a month of sample collection. MMSE and MIBG data were not available for all patients (N = 25 in MMSE, N = 28 in MIBG), because those procedures had been performed for clinical purposes. All data were collected from March 2010 to July 2016.

### Immunoassay protocol

Serum levels of theophylline were measured using the ARCHITECT iTheophylline system assay (Abbott Laboratory, Wiesbaden, Germany), which is an in-vitro chemiluminescent microparticle immunoassay with high sensitivity. The protocol was conducted in accordance with the instrument manual. Briefly, samples, anti-theophylline-coated paramagnetic microparticles, and theophylline acridinium-labeled conjugate were combined to obtain a reaction mixture. The anti-theophylline-coated microparticles bind to theophylline present in the sample and to the theophylline acridinium-labeled conjugate. After washing, the pre-trigger solution containing 1.32% hydrogen peroxide and trigger solution containing 0.35 N sodium hydroxide are added to the reaction mixture. The resulting chemiluminescent reaction is measured in relative light units (RLUs). The theophylline levels were calculated indirectly using a standard curve generated by calibrators and RLUs.

### Statistical analysis

A comparison between the two independent groups was performed using the Mann-Whitney U test. Fisher’s exact test was used to evaluate the significance of categorical variables. Correlational analysis was conducted using Spearman’s rank correlation test. The level of significance was set at p < 0.05. All analyses were carried out using GraphPad Prism software (GraphPad Prism Version 6.0, GraphPad software, SanDiego, USA).

## Results

The demographic data are shown in Table 1. There was no significant difference in age or sex between the PD and control groups. The calibration assay to check quality performance of the measurement in our laboratory demonstrated excellent goodness of fit (100%) and high sensitivity (lower detection limit of the method: 0.05 μg/mL) corresponding to the data provided by the manufacturer. The precision assay also yielded a low intra- and inter-assay coefficient of variation (CV) (≤ 3.0%) and nearly perfect recovery rate even at a level of 1.2 μg/mL, being below one eighth of the minimal therapeutic concentration of this drug (S1 Fig). Those data suggested the accuracy of this measurement for individuals not being administered theophylline.

**Table 1:** Characteristics of patients with PD and disease controls.

	PD group (n = 31)	Control group (n = 33)
Age (years)	67 (41–81)	64 (16–84)
Sex (numbers of females)	8 [26%]	9 [27%]
H&Y stage		
Grade 1	4 [13%]	-
Grade 2	10 [32%]	-
Grade 3	14 [45%]	-
Grade 4	3 [10%]	-
UPDRS part III (points)	19 (5–55)	-
Duration from onset (months)	35 (8–224)	-
H/M ratio of MIBG		
Early phase	1.81 (1.26–2.98)	-
Delayed phase	1.46 (1.14–3.24)	-
MMSE (points)	29 (19–30)	-

Data for continuous variables are expressed as median values (maximum-minimum). The rows of the H&Y stage indicate numbers of patients in each grade. Percentages of the subjects in each group are presented in brackets.

**Fig 1:**
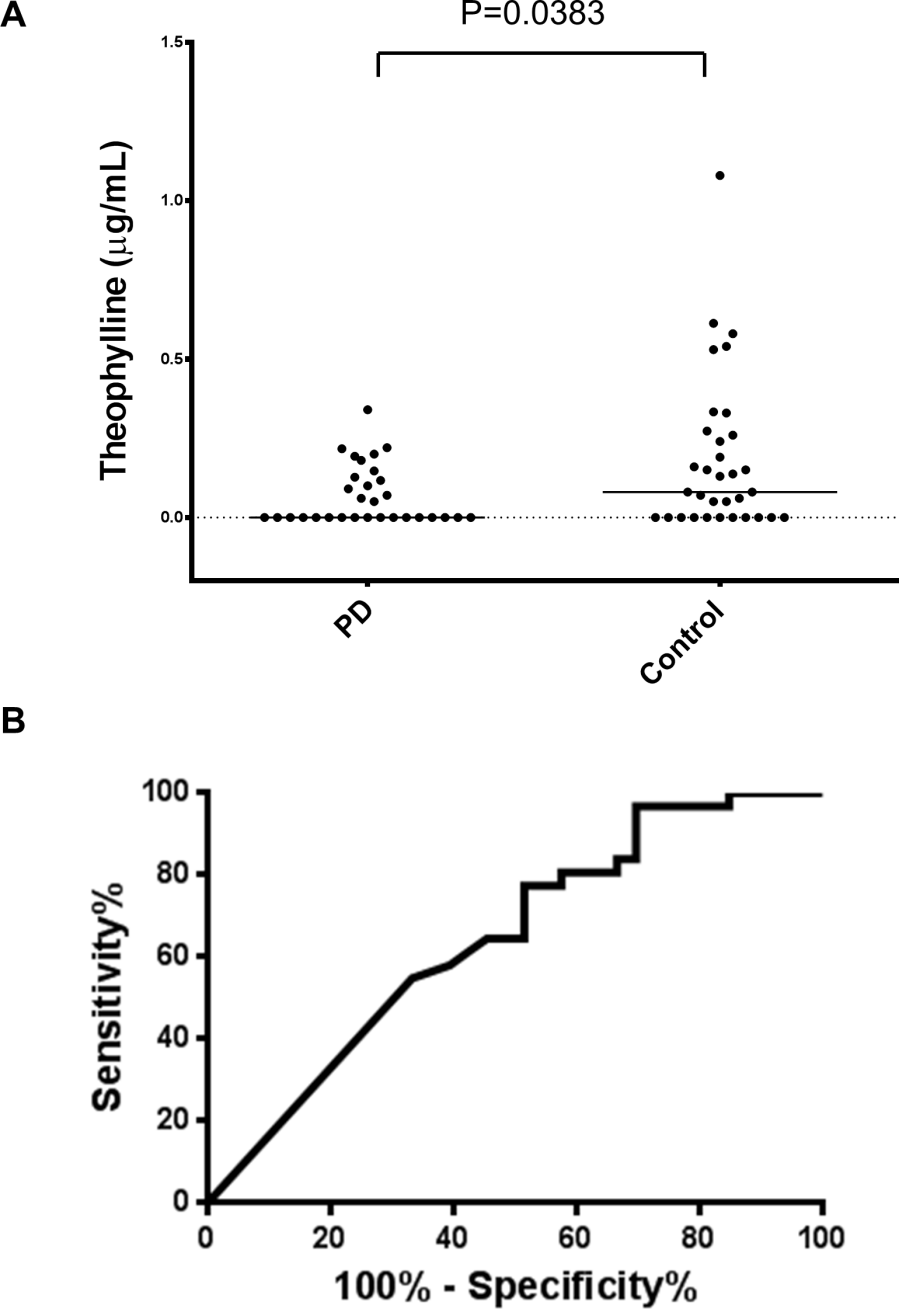
Theophylline levels in sera of the PD and control groups. Fig 1(A) Scatter plot showing the levels of serum theophylline in the control (n = 33) and PD (n = 31) groups. Bars indicate median values. The levels of theophylline in the PD group were significantly lower than those in the control group (P = 0.0383). Fig 1(B) ROC curve showing serum levels of theophylline to discriminate PD patients from controls. The AUC value was 0.65.

**Fig 2:**
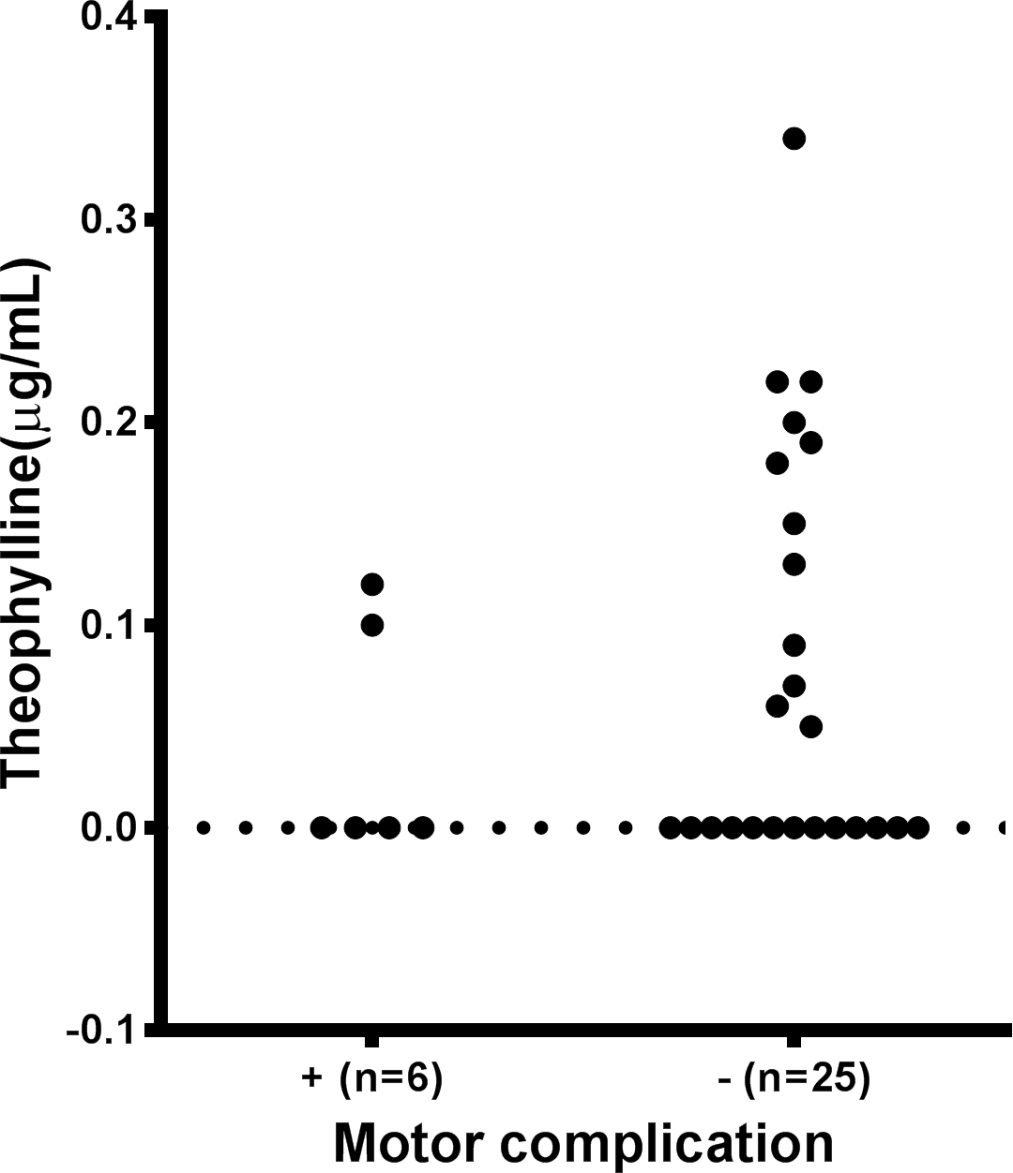
Serum levels of theophylline in PD patients with and without motor complications. Scatter plot showing levels of serum theophylline in patients with PD with (n = 6) and without (n = 25) motor complications. The levels of serum theophylline in patients with motor complications were lower than in those without them, although the difference was not significant (p = 0.42).

Serum levels of theophylline were detected in 45% of the PD group (14 of 31) and 67% of the control group (22 of 33). The serum levels of theophylline in the PD group were significantly lower than in the control group (PD: 0.07±0.09 μg/mL, control: 0.18±0.24 μg/mL, p = 0.0383, Fig 1A). There was no significant difference in serum levels of theophylline between males and females in our cohort, even though serum caffeine and estrogen share a common metabolic pathway of cytochrome P450 [11]. The median value of the serum theophylline levels was lower in the PD group than in the control group, even when males and females were separately analyzed, although the trend did not reach significance in the males (S2 Fig). The area under the receiver operating characteristic curve was 0.65 (Fig 1B). Levels of serum theophylline in patients with motor complications were lower than in those without them (PD with motor complications: 0.04±0.05 μg/mL, PD without motor complications: 0.08±0.10 μg/mL), although the difference was not significant (p = 0.42) (Fig 2). Serum levels of theophylline in the PD group were not correlated with the H&Y stages, UPDRS-III scores, or H/M ratio of the MIBG uptake (S3 Fig). Serum levels of theophylline in the PD group were not correlated with the duration from onset or MMSE score. There was no significant correlation between the serum theophylline levels and age in any group (S4 Fig).

## Discussion

The present study demonstrated that serum levels of theophylline measured by a commercially available chemiluminescent microparticle immunoassay were significantly lower in the PD group than control group. Serum theophylline levels in the PD group were not correlated with the age, duration from onset, severity of the disease, or H/M ratio of the MIBG uptake. Those results were consistent with the previous reports of Hatano et al. [6] and Fujimaki et al. [8].

Caffeine and its metabolites classified as methylxanthines are non-selective adenosine receptor antagonists, antagonizing A_1_, A_2_, and A_3_ receptors [12]. The following epidemiological, animal experimental, and clinicopharmacological observations have provided supporting evidence that they play important roles in PD pathogenesis and consequently, their levels could serve as diagnostic biomarkers of early as well as preclinical PD. First, caffeine consumption was an epidemiologically established protective factor against not only the development but also progression of PD [13–16]. Second, caffeine and its metabolites attenuated dopaminergic neuronal degeneration in animal models of PD treated with 1-methyl-4-phenyl-1,2,3,6-tetrahydropyridine, owing to the blockade of adenosine A2A receptors [17–19]. Third, a selective adenosine A2A receptor antagonist of istradefylline improved the motor symptoms of patients with PD [20]. A similar beneficial effect was also noted with caffeine [21].

**Fig 3:**
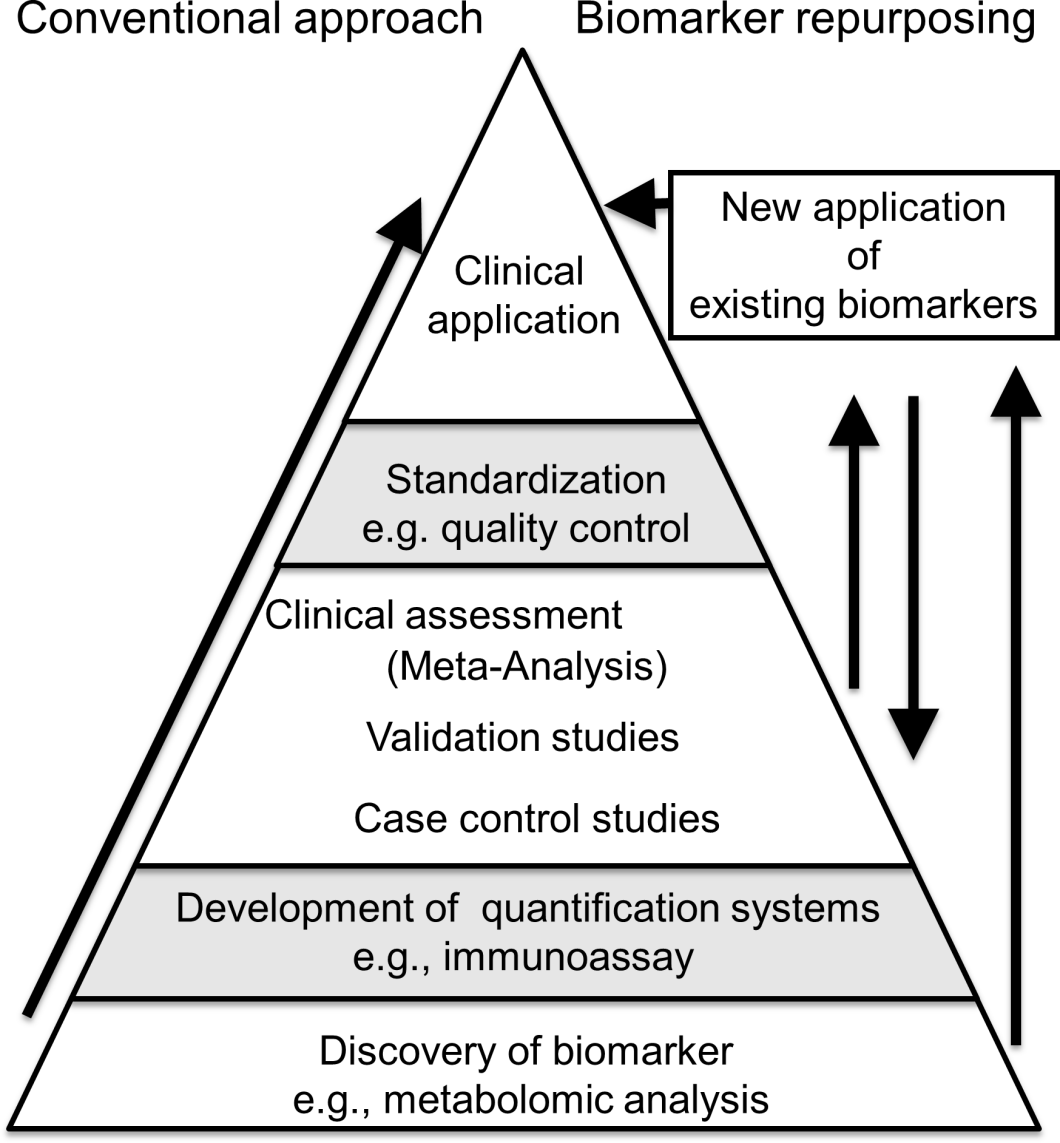
Concept of biomarker repurposing. The process of biomarker development is shown. Biomarker repurposing, the novel application of existing biomarkers for other purposes, enables the skipping of the development of quantification and standardization processes [27, 28].

The ARCHITECT iTheophylline used in the study is designed to have an assay precision of ≤ 10% intra- and inter-assay coefficient variance concordance, according to the instruction manual provided by the manufacturer [22]. Our data corresponded with theirs even at levels lower than the minimal therapeutic concentration of theophylline. Although serum theophylline could not be measured in all samples due to the detection limit of the system, such an analytical limitation would be overcome in the future by the introduction of automated ultrasensitive immunoassay techniques, e.g., single molecular array technology and a superconducting quantum interference device-based immuno-magnetic analyzer [23]. Besides its cost-effectiveness, this system has the marked advantage that it is already globally available in clinical laboratories and has been proven to show excellent inter-laboratory concordance (%CV≤5%) on external quality assessments [24]. Such international quality control studies of biomarkers have often faced difficulties in the presence of neurodegenerative diseases due to marked variability in the observed levels of candidate biomarkers. In fact, the international standardization projects on CSF biomarkers for AD and PD fields both failed to reach a consensus on universal cutoff values due to inter-laboratory variability [25, 26]. Our approach of applying known medical biomarkers (i.e., a therapeutic drug monitoring system for theophylline) to other purposes (i.e., biomarker of PD) might be similar to the so-called “drug repurposing” in pharmaceutical science from the perspective that this approach allows us to skip the process from the biomarker discovery phase to clinical application (Fig 3). Subsequently, it would contribute to shortening the period of time needed for biomarker development.

We acknowledge that the small sample size was a major limitation of the study. This may have weakened the statistical power, possibly leading to the result that the difference in theophylline levels between PD groups with and without motor complications failed to reach significance [8]. The lack of data on caffeine intake was another limitation. We collected the samples after three-hour fasting, while there was no restriction of tea or coffee intake before sampling. Despite the weak influence of the caffeine load on serum theophylline levels [9], we cannot rule out the possibility that our data may have been affected by caffeine consumption directly prior to sampling. In the future, case-control studies involving sufficient numbers of participants with information on caffeine consumption as well as international quality control programs are needed to confirm our findings and promote the clinical application of this method.

## Conclusions

Theophylline levels were measurable in serum using a conventional drug monitoring system and were significantly lower in the PD compared with control group. This immunoassay method has the potential to offer a reliable biomarker of this disease with the advantages of a markedly standardized and cost-effective protocol.

## Supporting information

S1 Fig The calibration assay for quality control of the measurement by the ARCHITECT iTheophylline system assay. The standard curve is shown in the figure. The inter-assay CV was 0.3% at a low concentration (5 μg/mL), 2.93% at an intermediate concentration (12 ug/mL), and 0.32% at a high concentration (24 ng/mL) (n = 2). The intra-assay CV was 2.01% at a low concentration (5 μg/mL), 0.39% at an intermediate concentration (2 μg/mL), and 1.6% at a high concentration (24 μg/mL) (n = 3). Furthermore, we diluted the intermediate calibrator 10-fold and 100-fold in saline and measured each solution using the kit. The inter-assay CV was 0.8% at the concentration diluted 10-fold (1.2 μg/mL) and 33.3% at that diluted 100-fold (0.12 μg/mL) (n = 2). The intra-assay CV was 2.0% at a concentration of 1.2 μg/mL and 5.1% μg/mL at a concentration of 0.12 μg/mL (n = 2). The percent recovery of the intermediate calibrator was 100% at 1.2 μg/mL and 91% at 0.12 μg/mL.
(TIF)

S2 Fig **Theophylline levels in sera of PD (A) and control (B) groups were compared between males and females. Theophylline levels in sera of males (C) and females (D) were compared between PD and control groups.** There was no significant difference between the sexes. The median serum theophylline levels were higher in the PD group in both sexes, although the trend did not reach significance. Bars indicate median values.
(TIF)

S3 Fig **The association between serum levels of theophylline and UPDRS-III scores (A), H&Y stages (B), and the H/M ratio in the early (C) and delayed (D) phases in the MIBG myocardial images of the PD group.** There was no significant correlation between them.
(TIF)

S4 Fig **The association between serum levels of theophylline and the duration from onset (A) MMSE scores (B), and ages (C) in the PD group. The association between serum levels of theophylline and age in the control group (D)**. There was no significant correlation between them.
(TIF)
