# Supplementary figures and images for: Biomarker repurposing: Therapeutic drug monitoring of serum theophylline offers a potential diagnostic biomarker of Parkinson’s disease
Source: PLoS One. 2018 Jul 25;13(7):e0201260. doi: 10.1371/journal.pone.0201260 (PMC6059449; doi:10.1371/journal.pone.0201260)

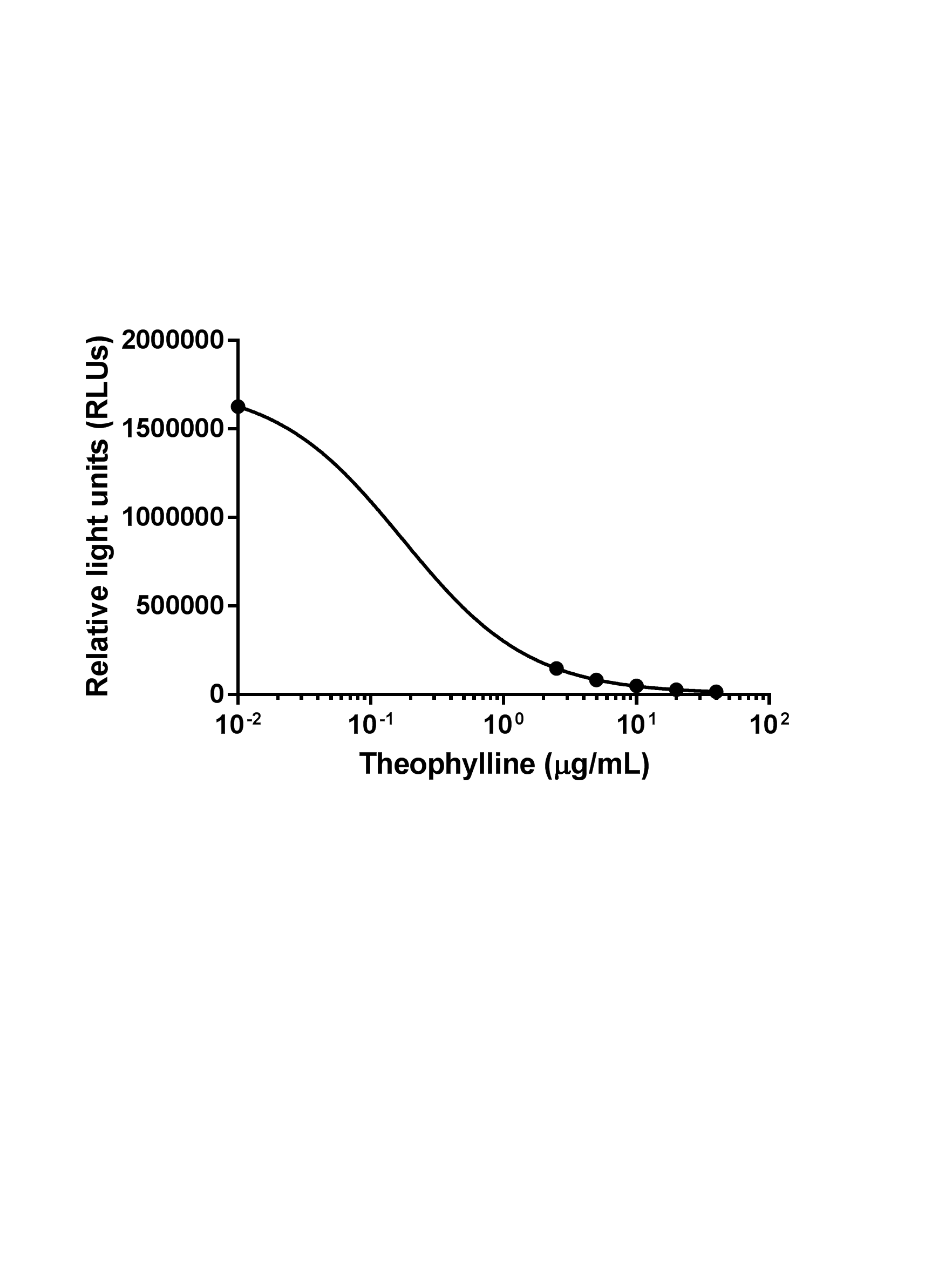

Supplement: S1 Fig — The standard curve is shown in the figure. The inter-assay CV was 0.3% at a low concentration (5 μg/mL), 2.93% at an intermediate concentration (12 ug/mL), and 0.32% at a high concentration (24 ng/mL) (n = 2). The intra-assay CV was 2.01% at a low concentration (5 μg/mL), 0.39% at an intermediate concentration (2 μg/mL), and 1.6% at a high concentration (24 μg/mL) (n = 3). Furthermore, we diluted the intermediate calibrator 10-fold and 100-fold in saline and measured each solution using the kit. The inter-assay CV was 0.8% at the concentration diluted 10-fold (1.2 μg/mL) and 33.3% at that diluted 100-fold (0.12 μg/mL) (n = 2). The intra-assay CV was 2.0% at a concentration of 1.2 μg/mL and 5.1% μg/mL at a concentration of 0.12 μg/mL (n = 2). The percent recovery of the intermediate calibrator was 100% at 1.2 μg/mL and 91% at 0.12 μg/mL. (TIF) [file pone.0201260.s001.TIF]

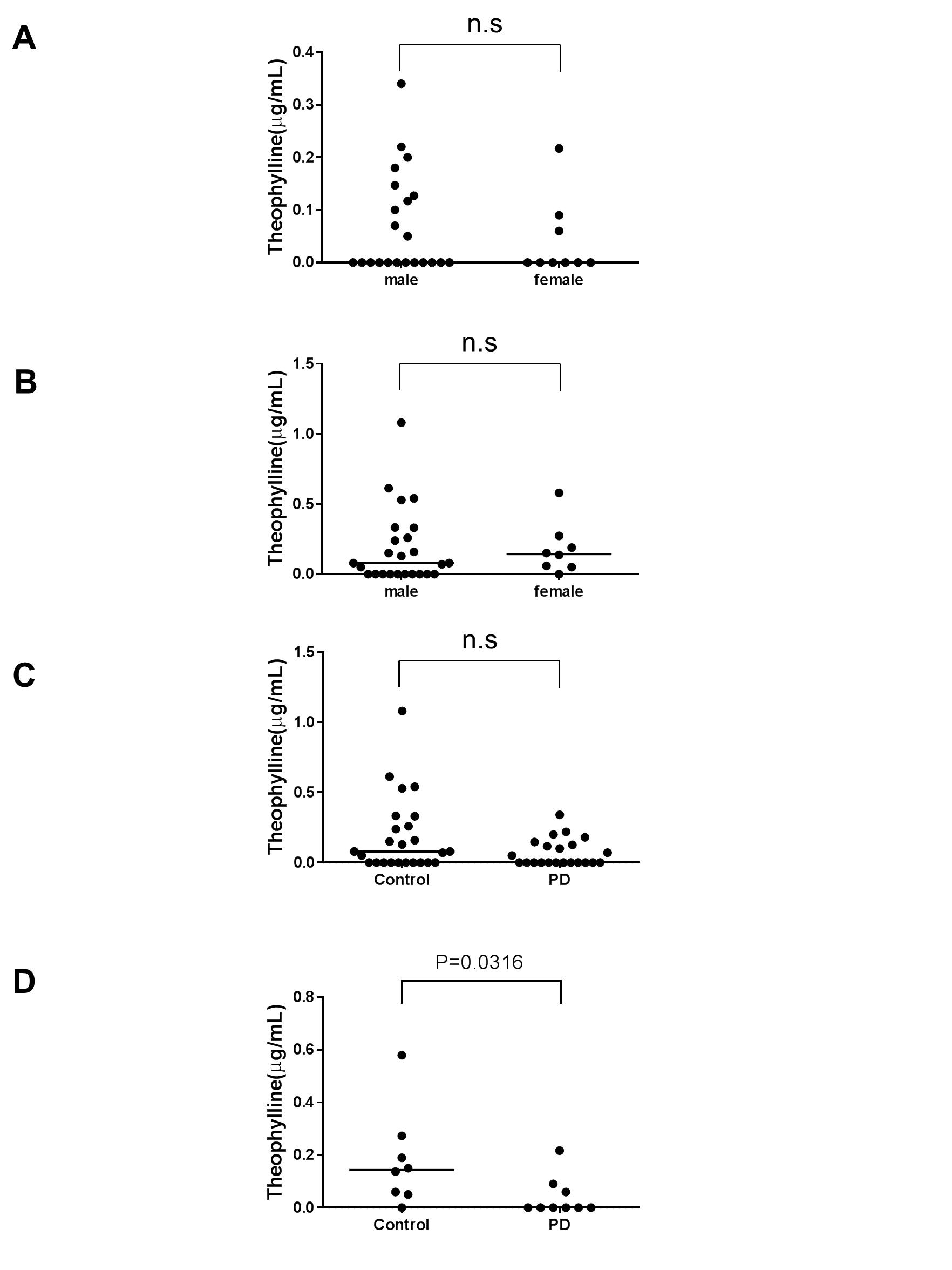

Supplement: S2 Fig — Theophylline levels in sera of PD (A) and control (B) groups were compared between males and females. Theophylline levels in sera of males (C) and females (D) were compared between PD and control groups. There was no significant difference between the sexes. The median serum theophylline levels were higher in the PD group in both sexes, although the trend did not reach significance. Bars indicate median values. (TIF) [file pone.0201260.s002.TIF]

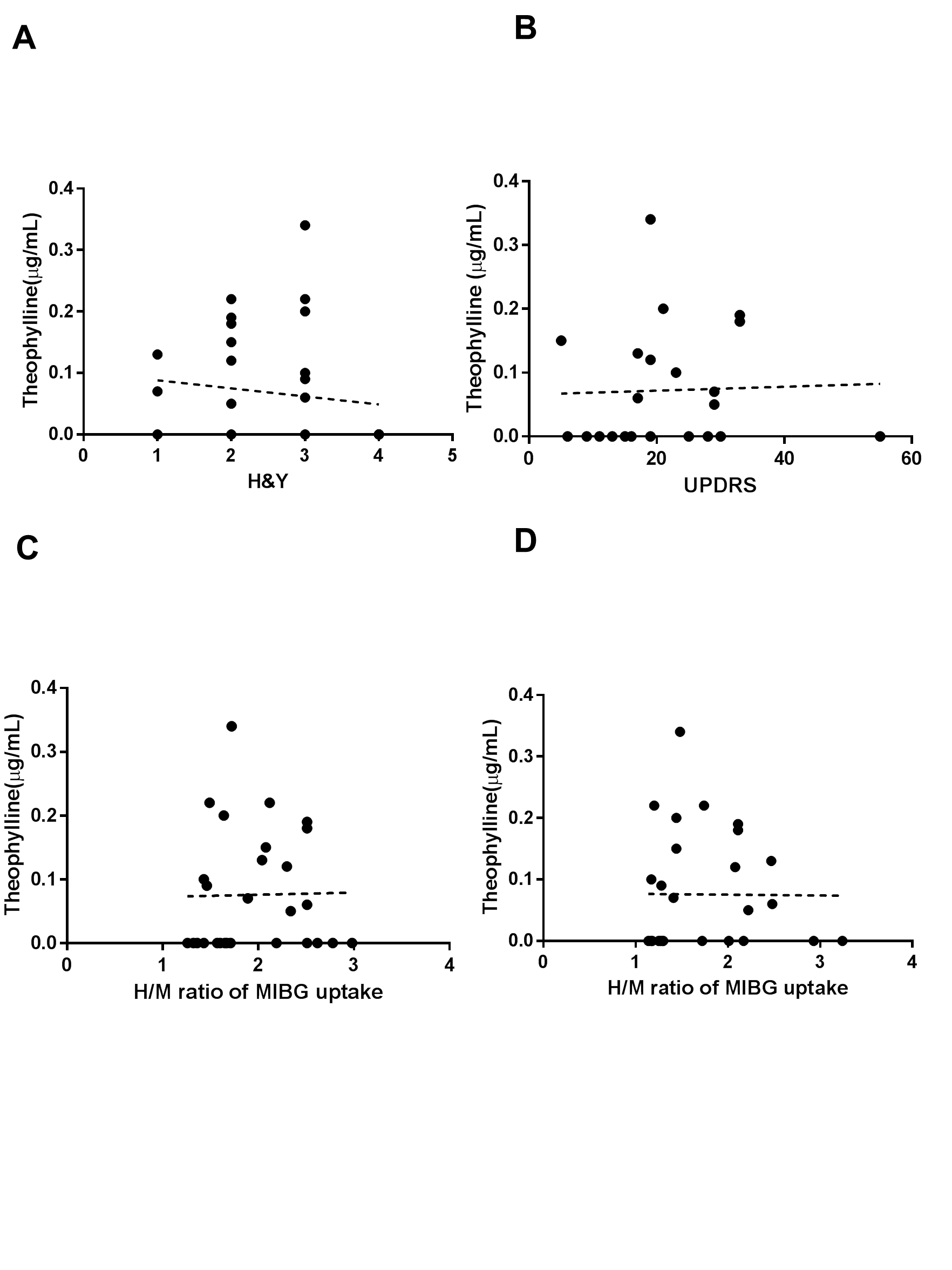

Supplement: S3 Fig — The association between serum levels of theophylline and UPDRS-III scores (A), H&Y stages (B), and the H/M ratio in the early (C) and delayed (D) phases in the MIBG myocardial images of the PD group. There was no significant correlation between them. (TIF) [file pone.0201260.s003.TIF]

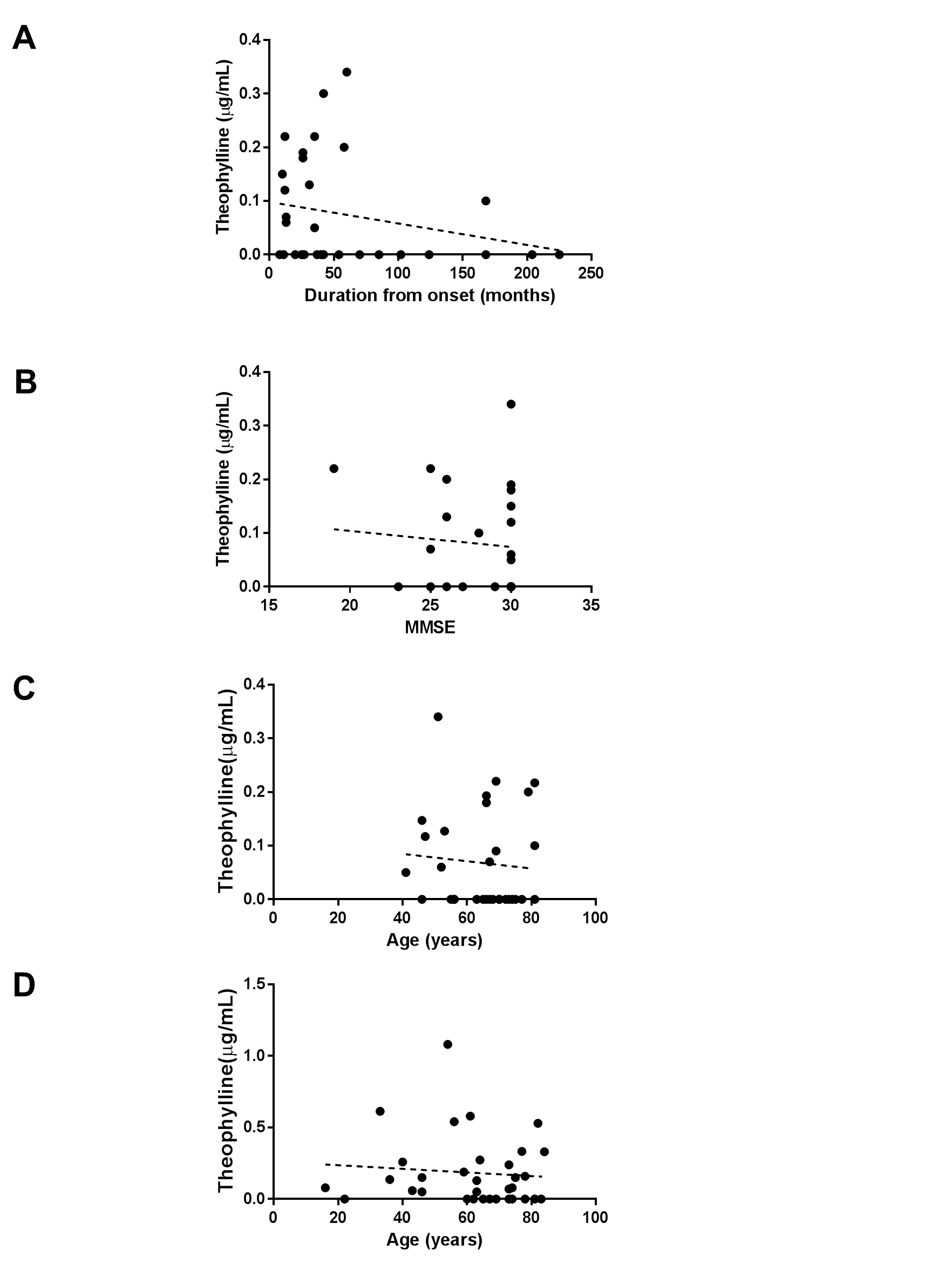

Supplement: S4 Fig — The association between serum levels of theophylline and the duration from onset (A) MMSE scores (B), and ages (C) in the PD group. The association between serum levels of theophylline and age in the control group (D). There was no significant correlation between them. (TIF) [file pone.0201260.s004.TIF]
